# S100A11 Promotes Metastasis via AKT and ERK Signaling Pathways and Has a Diagnostic Role in Hepatocellular Carcinoma

**DOI:** 10.7150/ijms.80503

**Published:** 2023-01-31

**Authors:** Mei Zheng, Huan Meng, Yunhui Li, Jingren Shi, Ying Han, Changxu Zhao, Jin Chen, Jinyu Han, Jing Liang, Yuan Chen, Qiqi Liu, Yajie Wang

**Affiliations:** 1 Department of Clinical Laboratory, Beijing Ditan Hospital, Capital Medical University, Beijing, 100015, China.; 2 Beijing Institute of Microbiology and Epidemiology, Beijing, 100850, China.

**Keywords:** S100A11, hepatocellular carcinoma, biomarker, metastasis, signaling pathway

## Abstract

Hepatocellular carcinoma (HCC) is the most common and malignant liver tumor worldwide, although the treatment approaches for HCC continue to evolve, metastasis is the main reason for high mortality rates. S100 calcium-binding protein A11 (S100A11), an important member of the S100 family of small calcium-binding proteins, is overexpressed in various cells and regulates tumor development and metastasis. However, few studies report the role and underlying regulatory mechanisms of S100A11 in HCC development and metastasis. Herein, we discovered that S100A11 is overexpressed and associated with poor clinical outcomes in HCC cohorts, and we provided the first demonstration that S100A11 could serve as a novel diagnostic biomarker used in conjunction with AFP for HCC. Further analysis implied that S100A11 outperforms AFP in determining whether HCC patients have hematogenous metastasis or not. Using *in vitro* cell culture model, we demonstrated that S100A11 is overexpressed in metastatic hepatoma cells, knockdown of S100A11 decreases hepatoma cells proliferation, migration, invasion, and epithelial-mesenchymal transition process by inhibiting AKT and ERK signaling pathways. Altogether, our study provides new sights into the biological function and mechanisms underlying S100A11 in promoting metastasis of HCC and explores a novel target for HCC diagnosis and treatment.

## Introduction

Primary liver cancer (PLC) is the sixth most common cancer and the third leading cause of cancer-related death worldwide, posing a serious threat to human health and the world economy 1, 2. The most frequent type of PLC is hepatocellular carcinoma (HCC), which accounts for 90% of all PLC worldwide, followed by cholangiocarcinoma 3. Major factors for HCC include hepatitis B or C virus (HBV/HCV) infection, chronic hepatitis, cirrhosis, aflatoxin or alcohol exposure, etc. Among them, chronic HBV infection is the most common cause of HCC in China 3-5. Regrettably, most HCC patients are diagnosed at advanced disease stages due to its insidious onset 6. Although treatment options continue to evolve, metastasis is the primary cause of its high mortality and the critical challenge in HCC therapeutics 7-9. The main routes of HCC metastasis are hematogenous metastasis, lymph node metastasis, direct infiltration, etc. Accordingly, discovering diagnostic and prognostic biomarkers and exploring the mechanisms underlying HCC progression and metastasis will bring new perspectives to the treatment approaches.

S100 calcium-binding protein A11 (S100A11), also known as calgizzarin, S100C, is an important member of the S100 protein family with two EF-hand motifs 10, 11. It is a small molecular weight protein (almost 12kDa) and performs important functions inside and outside of cells, such as cell cycle, cell apoptosis, cell proliferation, cell migration, and cell invasion 10, 12, 13. Also, it plays a pivotal role in the epithelial-mesenchymal transition (EMT) process 14-16. Several studies showed that S100A11 is overexpressed in various malignant tumors, such as lung cancer 17, gastric cancer 18, cervical squamous cell carcinoma 19, breast cancer 20, glioblastoma 14, etc. However, it plays a tumor-suppressive role in bladder cancer 21, indicating that S100A11 has a double effect on regulation of cancers. Since it is poorly expressed in healthy livers, research into the role of S100A11 in liver disease is capturing growing attention in recent years. A few studies showed that S100A11 plays important roles in promoting hepatic steatosis via the RAGE-mediated AKT-mTOR signaling pathway, FOXO1-mediated autophagy, and lipogenesis 22, 23. Tingting Zhu et al. highlighted that S100A11 promotes liver fibrogenesis by targeting the TGF-β signaling pathway and can be a potential biomarker for liver fibrosis 24. Besides, previous studies demonstrated that S100A11 promotes cell proliferation in intrahepatic cholangiocarcinoma 25. However, it remains unknown whether S100A11 can present as a circulating biomarker for HCC. As well, the detailed function and underlying biological molecular mechanisms of S100A11 in the progression and metastasis of HCC are poorly understood.

With the aim to identify the role of S100A11 in HCC, we sought to offer the first confirmation of S100A11 expression and clinical significance of S100A11 in HCC patients. Herein, we found that S100A11 is overexpressed and associated with unfavorable clinical outcomes in HCC cohorts. Further studies demonstrated that S100A11 is overexpressed in metastatic hepatoma cells, and S100A11 knockdown decreases hepatoma cells proliferation, migration, invasion and EMT progress by inhibiting AKT and ERK signaling pathways. Our study reveals that S100A11 has a diagnostic role and promotes metastasis in HCC.

## Materials and methods

### Cell lines and culture

Hep3B, HepG2, and Li-7 cells were purchased from the Cell Culture Center of the Institute of Basic Medical Sciences, Chinese Academy of Sciences (Beijing, China), HCCLM3 cell was purchased from the China Center for Type Culture Collection of Wuhan University (Wuhan, China). All cells were tested for Mycoplasma contamination and verified by STR analysis. HepG2, Hep3B, and HCCLM3 were cultured in Dulbecco's modified Eagle's medium (DMEM) with 10% fetal bovine serum (FBS), the Li-7 cell was cultured in RPMI-1640 with 10% FBS, the stable cell lines were maintained in DMEM (HCCLM3-sh-con, HCCLM3-sh-S100A11) or RPMI-1640 (Li-7-sh-con, Li-7-sh-S100A11) containing 10% FBS and 2 ~ 4 μg/mL puromycin. All cells were maintained in a cell incubator (37 °C) with 5% CO2.

### Patients and plasma

HCC and control specimens were collected at Beijing Ditan Hospital, Capital Medical University (Beijing, China). We enrolled 34 patients with HCC and 22 healthy control subjects. The control group includes individuals who received physical examination or sought medical attention for toothache, headache, or other mild symptoms. All of the control subjects tested negative for HBV infections and AFP. The clinical information was summarized in Table 1. As this trial used patients' residual serum after daily detection, no extra collection was required, and patient privacy and secret information were protected, no harm to the subjects was envisaged. So, a waiver of informed consent was obtained before plasma collection for this study. The study was approved by the ethical committee of Beijing Ditan Hospital (2020-025-01) and conducted following the 1991 Declaration of Helsinki.

### Antibody and reagents

The antibodies that we used for the Western Blot assay are as follows: Primary antibody: anti-S100A11 (10237-1-AP) was purchased from Proteintech (USA), anti-GAPDH (P04406), anti-E-cadherin(#14472), anti-ERK (#4695T), anti-p-ERK (#4370T), anti-AKT (#5691S) and anti-p-AKT (#4060S) were from Cell Signaling Technology (Shanghai, China), anti-N-cadherin (ab76011) was from Abcam company (MA, United States); Secondary antibodies: anti-mouse IgG, HRP-linked antibody(#7076) and mouse anti-rabbit IgG mAb (HRP conjugate) were also purchased from Cell Signaling Technology (Shanghai, China). PrimScript RT reagent Kit with gDNA Eraser (RR047A) was from Takara (Japan), and SYBR Green PCR Master Mix was from Thermo Fisher Scientific (USA).

### RT-qPCR

To detect differences in S100A11, E-cadherin or N-cadherin mRNA expression, total cell RNA was extracted from hepatoma cell lines according to the manufacturer's instructions. Up to 1 μg RNA was used to perform reverse transcription using PrimeScript RT reagent Kit with gDNA Eraser. The qPCR was performed using SYBR Green PCR Master Mix and the Applied Biosystems (ABI) 7500 Real-Time PCR system. The relative amounts of S100A11, E-cadherin, and N-cadherin were calculated by the contrastive Ct method (2^^-ΔΔCT^). The mRNA expression of the human housekeeping gene GAPDH was used as an endogenous control. Primer sequences for qPCR are as follows:

- GAPDH-Forward: GGCCTCCAAGGAGTAAGACC;
- GAPDH-Reverse: GATTCAGTGTGGTGGGGGAC;
- S100A11-Forward: GCTGCCTTCACAAAGAACCA;
- S100A11-Reverse: AAGCCATAGCTAGGCCACCA;
- E-cadherin-Forward: GAGTGCCAACTGGACCATTCAGTA;
- E-cadherin-Reverse: AGTCACCCACCTCTAAGGCCATC;
- N-cadherin-Forward: GTCAGCAGAAGTTGAAGAAATAGTG;
- N-cadherin-Reverse: GCAAGTTGATTGGAGGGATG.

### Western Blot assay

The total protein of cells was extracted by incubating with RIPA buffer containing proteinase and phosphatase inhibitors. After centrifuging at 12,000g, 4 °C for 5 min, the supernatants were boiled with loading buffer at 100 °C for 10 min. The protein was separated by 4~12% SDS-PAGE and then transferred to a PVDF membrane. Blocking by TBST with 5% milk or BSA, the membrane was incubated with the first antibody at 4 °C overnight. After rinsing in TBST, the membrane was incubated with an appropriate secondary antibody for 1h at room temperature, then exposed to ECL reagent after rinsing in TBST.

### CCK8 assay

Cell viability was measured by CCK8 kit (Dojindo, Japan) according to manufacturer's instructions. Cells were plated in five 96-well plates at a density of 2,000 cells/well. After cell attachment, replaced the culture medium by DMEM with 10% CCK8, 2h later, measuring the absorbance values at OD 450nm. Twenty-four hours, 48h, 72h, and 96h after cell seeding, repeating the above CCK8 detection procedure.

### Cell clone formation assay

A total of 2,000 cells were seeded in triplicates into 6-well plates and cultured for 2 weeks. Changing fresh culture medium every 3 days for this period. Two weeks later, the cells were washed with PBS three times and stained with 4% paraformaldehyde for 20 min at room temperature. Then, cells were stained with 0.1% crystal violet, followed by washing with PBS and photographing.

### EdU incorporation assay

The EdU incorporation assay was performed by BeyoClick™ EdU Cell Proliferation Kit with Alexa Fluor 594. A total of 2,000 cells were seeded in a 96-well plate and cultured in a cell incubator. After 48h, cells were incubated with EdU for 2h and then washed three times in PBS, fixed in 4% paraformaldehyde for 15 min, and permeabilized with 0.3% Triton X-100 for 15 min. Finally, EdU detection was carried out according to the manufacturer's instructions.

### Transwell assay

The Transwell invasion or migration assays were conducted using Transwell chambers coated with or without Matrigel. Cells in 200μl medium without FBS were seeded in the upper chamber and the bottom chamber was filled with 600μl of medium containing 20% FBS. The invasive or migrative cells were fixed with 4% paraformaldehyde after 48h of incubation and stained with 0.1% crystal violet. Subsequently, washing the chamber three times with PBS and wiping cells in the upper chamber with a moistened cotton swab, and obtained the images under a microscope at ×400 magnification.

### ELISA assay

Before the experiments start, bring all samples and kit components to room temperature. S100A11 levels in human plasma were determined by an S100A11 ELISA kit (R&D Systems). The optical density of samples was read at wavelengths of 450 nm. According to the standard curve, S100A11 concentration of each sample was obtained.

### Statistical analysis

Statistical analysis was performed using GraphPad Prism 8 and IBM SPSS Statistics 25. The differences between the two groups were calculated by student's t-test if continuous variables fit a normal distribution. Non-parametric tests were used if data did not follow a normal distribution. All data were presented as mean ± SD. *P* < 0.05 indicated that the difference was statistically significant.

## Results

### S100A11 is overexpressed and associated with poor survival in HCC cohorts

**Figure 1:**
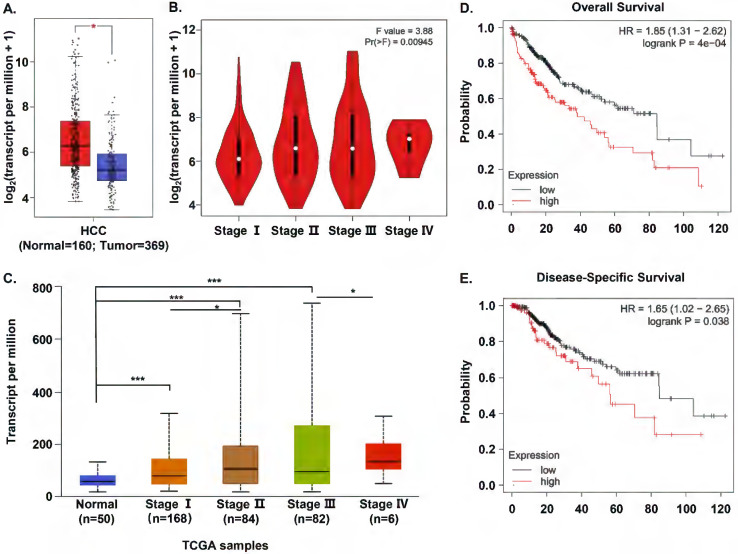
** S100A11 is overexpressed and associated with poor survival in HCC cohorts. (A)** The differential expression of S100A11 between tissues of HCC (n=369) and normal controls (n=160) based on data available from TCGA and GTEx database. **(B)** Analysis of mRNA expression of S100A11 in different pathological stages by using GEPIA database. **(C)** Expression of S100A11 in HCC based on individual cancer stages using UALCAN database. **(D, E)** The correlation of S100A11 expression with OS (D) and DSS (E) time in HCC samples using the Kaplan-Meier plotter. * P < 0.05, *** P < 0.001.

In order to evaluate S100A11 expression in HCC patients, we compared S100A11 mRNA expression between human HCC and normal samples based on the RNA-seq data available from TCGA and GTEx database using Gene Expression Profiling Interactive Analysis (GEPIA). We found that S100A11 mRNA expression was significantly overexpressed in HCC samples than in normal tissues (Figure 1A). Then, we analyzed the association between the expression of S100A11 and the pathological stage in HCC samples. The results showed that S100A11 displayed a positive correlation with the pathological stage (Figure 1B). To verify the data, we chose another online public database, UALCAN, to validate the mRNA expression of S100A11 in normal samples and different stages of HCC samples based on TCGA data. And the results were consistent with that in GEPIA database (Figure 1C). To assess the prognostic value of S100A11 for HCC patients, we used the Kaplan-Meier plotter to analyze the correlations of S100A11 expression with overall survival (OS) and disease-specific survival (DSS) time. The results implied that high expression of S100A11 was associated with a shorter OS (Figure 1D) and DSS time (Figure 1E). To summarize, these data suggest S100A11 is overexpressed in HCC patients and can be an effective predictor for survival prognosis.

### S100A11 may present as a promising biomarker in HCC patients

**Figure 2:**
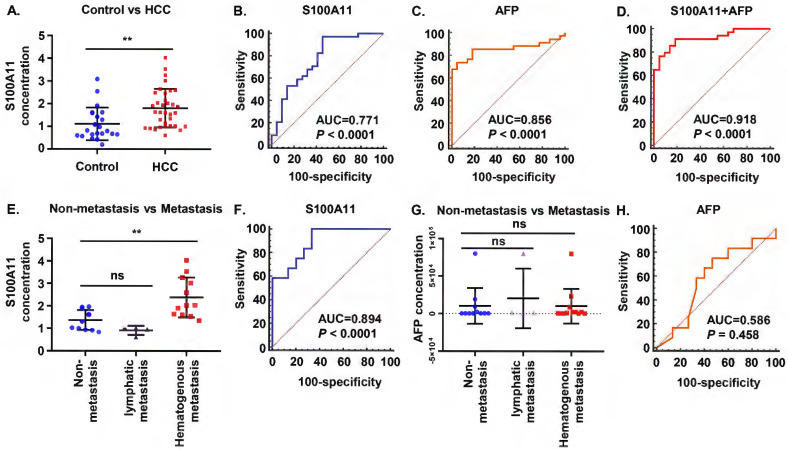
** S100A11 may present as a promising biomarker in HCC patients. (A)** Plasma levels of S100A11 in healthy controls (n = 22) and HCC patients (n = 34). **(B-D)** ROC curves of plasma S100A11(B), AFP (C), and S100A11+AFP (D) for HCC patients. **(E)** Plasma levels of S100A11 in male patients with non-metastasis (n = 11), lymphatic metastasis (n = 4), and hematogenous metastasis (n = 12).** (F)** ROC curves of plasma S100A11 for hematogenous metastasis in male HCC patients. **(G)** Plasma levels of AFP in male patients with non-metastasis (n = 11), lymphatic metastasis (n = 4), and hematogenous metastasis (n = 12). **(H)** ROC curves of AFP for hematogenous metastasis in male HCC patients. ns: no significance, ** P < 0.01.

Subsequently, we attempted to validate our findings in clinical samples. A total of 56 plasma samples, including 34 HCC patients and 22 normal controls, were identified. Results of the ELISA assay showed that plasma S100A11 was higher expressed in HCC patients than that in healthy control group (Figure 2A). To compare the predictive ability between S100A11 and alpha-fetoprotein (AFP) in discriminating HCC and normal controls, receiver operating characteristic (ROC) curves were calculated. The area under curve (AUC) of S100A11 for the predictive performance was 0.771 (95% CI: 0.640-0.873, *P <* 0.0001) (Figure 2B), and that of AFP was 0.856 (95% CI: 0.736-0.935, *P <* 0.0001) (Figure 2C), there was no statistically difference between two ROC curves (*P* = 0.3306 by two-sided Delong's test for correlated ROC curves). Interestingly, when using the best cutoff for S100A11 determined in the ROC curve (0.82 ng/mL) and the currently recommended clinical cutoff for AFP (20 ng/mL), we found that S100A11 has higher sensitivity but lower specificity, while AFP has higher specificity but lower sensitivity (Table 2). When used in a combination, optimal diagnostic performance (AUC: 0.918; sensitivity: 91.2%; specificity: 81.8%) was achieved (Figure 2D and table 2). As can be seen, S100A11 could be a promising non-invasive biomarker used in combination with AFP in HCC diagnosis.

Furthermore, we found that 16 out of 27 male patients (59.26%) had metastasis in our study, of whom 4 patients developed lymphatic metastasis and 12 patients developed hematogenous metastasis (mainly to lung and bone), while no metastasis was observed in female patients. So, further analysis on whether S100A11 is related to metastasis was performed on the male HCC cohorts. The results showed significantly higher S100A11 in patients with hematogenous metastasis than in patients with lymph node metastasis or without metastasis (Figure 2E). To assess the diagnostic efficacy of S100A11 in discriminating patients with hematogenous metastasis from individuals with non-hematogenous metastasis, ROC curve was analyzed. The results showed the AUC was 0.894 (95% CI: 0.716-0.979, *P<*0.0001) (Figure 2F), the specificity and sensitivity values were summarized in Table 2. However, in patients with no metastasis, lymphatic metastasis, and hematogenous metastasis, AFP levels showed no significant difference and no diagnostic power. (Figure 2G, H). These data suggest that S100A11 might be a potential prognostic indicator of hematogenous metastasis in HCC patients.

### S100A11 is overexpressed in metastatic hepatoma cells and is mainly localized in the cytoplasm

**Figure 3:**
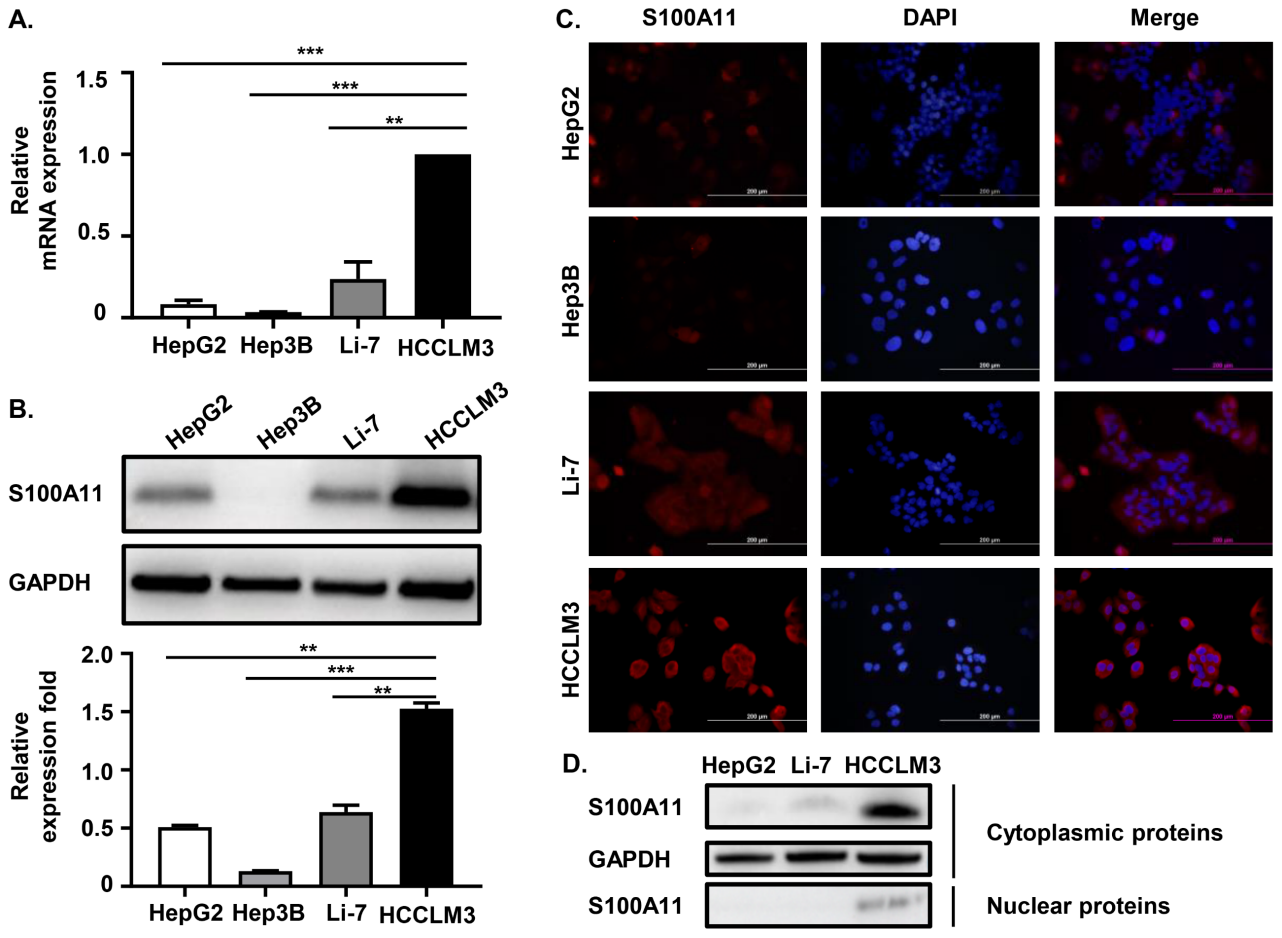
** S100A11 is overexpressed in metastatic hepatoma cells and is mainly localized in the cytoplasm. (A)** The mRNA expression level of S100A11 in HepG2, Hep3B, Li-7, and HCCLM3 cells determined by RT-qPCR. **(B)** The protein expression level of S100A11 in HepG2, Hep3B, Li-7, and HCCLM3 cells was determined by Western Blot assay. **(C)** The protein expression level and subcellular localization of S100A11 in HepG2, Hep3B, Li-7, and HCCLM3 cells determined by IFA. **(D)** The protein levels of S100A11 and GAPDH in cytoplasm and nucleus of HepG2, Li-7 and HCCLM3 cells determined by Western Blot assay. ** P < 0.05, *** P < 0.001.

We have proven that S100A11 is overexpressed in HCC patients and associated with hematogenous metastasis. To further investigate the detailed role of S100A11 in HCC progression and metastasis, we chose four different human hepatoma cell lines, including HCCLM3, HepG2, Hep3B, and Li-7, for further studies. Among them, HCCLM3 cell is highly metastatic. Firstly, we used RT-qPCR assay to detect S100A11 mRNA expression in these hepatoma cells, and we found that S100A11 mRNA in HCCLM3 was significantly overexpressed than the other three low-metastatic cell lines (Figure 3A). Western Blot assay got similar results (Figure 3B). Then, we also used immunofluorescence assay (IFA) to detect the expression level and subcellular localization of S100A11. The IFA results were consistent with the Western Blot results, as the level of S100A11 was highest in HCCLM3 and lowest in Hep3B. Besides, we found that S100A11 was mainly localized in the cytoplasm (Figure 3C). However, no difference in the subcellular localization characteristics of S100A11 was found in these hepatoma cell lines mentioned. Then, we extracted the nuclear and cytoplasmic proteins from HepG2, Li-7, and HCCLM3 cells, and detected S100A11 expression by Western Blot assay. Consistent with the IFA results, S100A11 was mostly detected in the cytoplasm of HCCLM3 with very little expression in the nucleus (Figure 3D). In HepG2 and Li-7 cells, S100A11 was only detected in the cytoplasm but not detected in the nucleus (Figure 3D), which may be related to the poor sensitivity of Western Blot assay. Collectively, the results described above imply that S100A11 is mainly localized in the cytoplasm with very small amount found in the nucleus, and might be involved in hepatoma cells metastasis.

### S100A11 participates in the regulation of the EMT process

**Figure 4:**
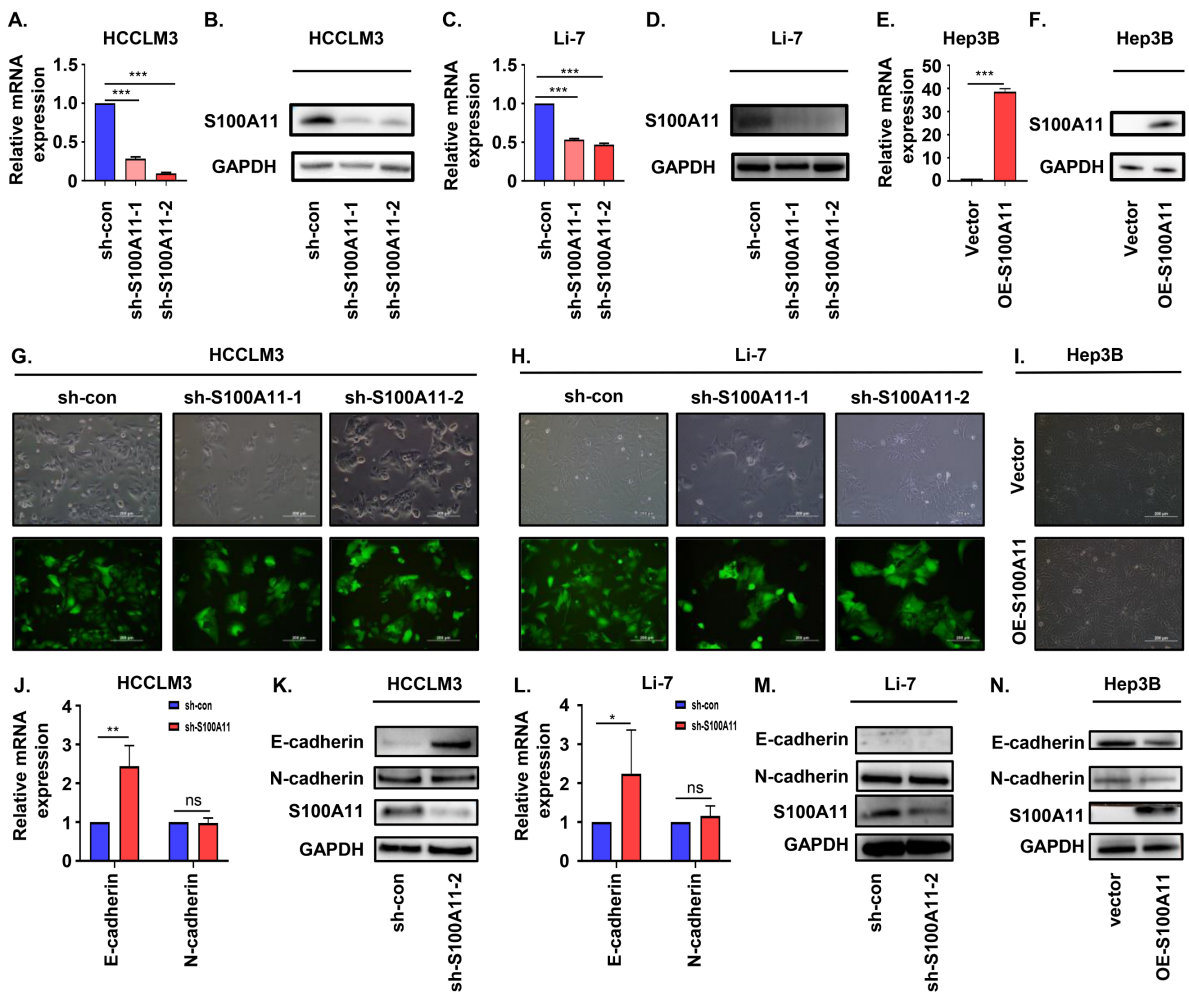
** S100A11 participates in the regulation of the EMT process. (A, C, E)** The mRNA expression level of S100A11 in HCCLM3-sh-con, HCCLM3-sh-S100A11, Li-7-sh-con, Li-7-sh-shS100A11, Hep3B-vector and Hep3B-OE-S100A11 cells. **(B, D, F)** The protein expression level of S100A11 in HCCLM3-sh-con, HCCLM3-sh-S100A11, Li-7-sh-con, Li-7-sh-S100A11, Hep3B-vector and Hep3B-OE-S100A11 cells. **(G-I)** Phase micrographs of HCCLM3-shcon, HCCLM3-sh-S100A11, Li-7-sh-con, Li-7-sh-S100A11 Hep3B-vector and Hep3B-OE-S100A11 cells. **(J, L)** The mRNA expression level of E-cadherin and N-cadherin in HCCLM3-sh-con, HCCLM3-sh-S100A11, Li-7-sh-con and Li-7-sh-S100A11 cells.** (K, M, N)** The protein expression level of E-cadherin and N-cadherin in HCCLM3-sh-con, HCCLM3-sh-S100A11, Li-7-sh-con, Li-7-sh-S100A11, Hep3B-vector and Hep3B-OE-S100A11 cells. ** P < 0.01, *** P < 0.001, ns: no significance.

To explore the role of S100A11 in liver cancer progression and metastasis, we used lentivirus expressing short hairpin RNA (shRNA) targeting scramble (sh-con) or S100A11 (sh-S100A11) to stably knockdown S100A11 expression in HCCLM3 and Li-7 cells, and conducted S100A11 overexpression (OE-S100A11) cells by transient transfection in Hep3B cells. The RT-qPCR results showed that S100A11 mRNA expression was significantly decreased in HCCLM3-shS100A11-1, 2 and Li-7-shS100A11-1, 2 compared to their negative control (Figure 4A, C) while increased in Hep3B-OE-S100A11 compared to Hep3B-vector (Figure 4E). Then, Western Blot assay was used to detect the protein expression of S100A11, and the results were consistent with the RT-qPCR results (Figure 4B, D, F). As described above, the S100A11 knockdown and overexpression cell models were established successfully.

Previous studies have reported that EMT plays an important role in cancer metastasis 26, 27. To evaluate whether S100A11 enhances EMT in hepatoma cells, we used optical microscopy to identify the morphology changes in S100A11 knockdown and overexpression cells. Herein, we showed that HCCLM3-sh-S100A11 and Li-7-sh-S100A11 cells grown in more dense colonies, and cell-cell adhesion was enhanced, while cells expressing scramble (control) shRNA were spindle-shaped and showed more dispersed growth characteristics (Figure 4G, H). However, the intercellular space in Hep3B-OE-S100A11 was slightly widened than that in Hep3B-vector cells (Figure 4I). These were typical morphological changes during EMT. E-cadherin and N-cadherin are crucial transmembrane glycoproteins involved in EMT process 27. To measure the expression of E-cadherin and N-cadherin following S100A11 knockdown and overexpression, RT-qPCR was conducted to detect E-cadherin gene expression and Western Blot assay was performed to detect protein expression. The RT-qPCR results showed that the relative mRNA expression of E-cadherin in HCCLM3-sh-S100A11and Li-7-sh-S100A11 was higher than that in their control cells. However, there was no change in N-cadherin expression upon S100A11 knockdown, which was not consistent with our speculation (Figure 4J, L). The Western Blot assay results showed that the protein expression of E-cadherin was significantly increased in HCCLM3-sh-S100A11 cells (Figure 4K) whereas decreased in Hep3B-OE-S100A11 cells (Figure 4N). Nonetheless, we did not detect E-cadherin protein expression in Li-7 cells (Figure 4M). However, the N-cadherin expression was not altered by either knockdown or overexpression of S100A11. The above results indicate that S100A11 could participate the EMT process in hepatoma cells, but the cause for this lack of difference in N-cadherin expression is unclear.

### Knockdown of S100A11 inhibits hepatoma cells proliferation, migration and invasion *in vitro*

**Figure 5:**
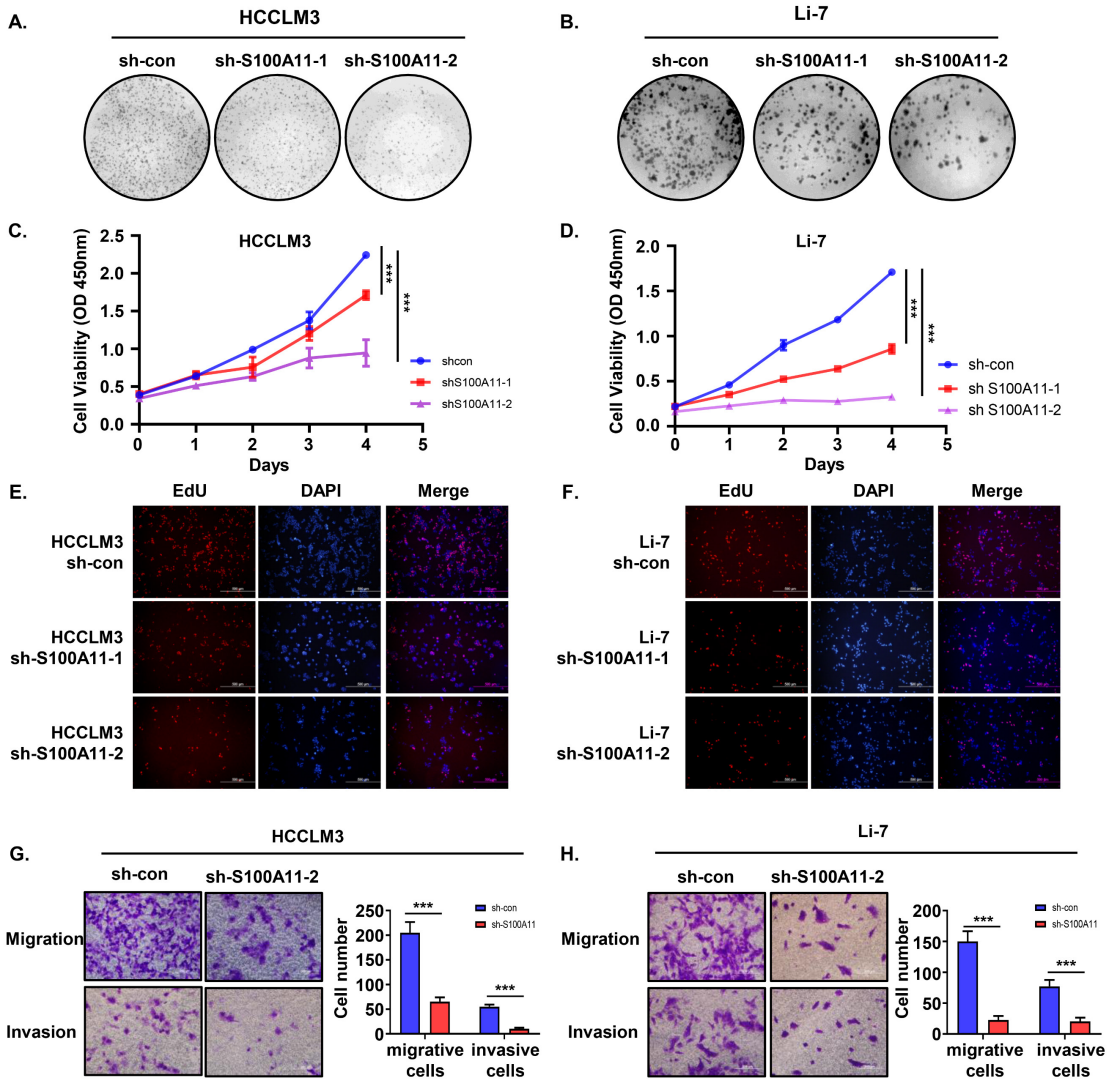
** S100A11 knockdown inhibits proliferation, migration, and invasion in hepatoma cells. (A, B)** Representative clone formation images of HCCLM3-sh-con, HCCLM3-sh-S100A11, Li-7- sh-con and Li-7-sh-S100A11 cells. **(C, D)** Assessment of the proliferation of HCCLM3-sh-con, HCCLM3-sh-S100A11, Li-7-sh-con, and Li-7-sh-S100A11 cells by CCK8 viability assay. **(E, F)** Assessment of the proliferation of HCCLM3-sh-con, HCCLM3-sh-S100A11, Li-7-sh-con, and Li-7-sh-S100A11 cells by EdU incorporation assay. **(G, H)** Assessment of the migration and invasion of HCCLM3-sh-con, HCCLM3-sh-S100A11, Li-7-sh-con, and Li-7-sh-S100A11 cells by Transwell assay. *** P < 0.001.

Cell proliferation is crucial for tumorigenesis and metastasis 28. We performed cell colony formation assay, CCK8 assay, and EdU incorporation experiment to explore the role of S100A11 on cell proliferation and viability. As indicated by the clone formation, there was a significant reduction in the number of colonies by S100A11 knockdown of HCCLM3 and Li-7 cells after 14 days (Figure 5A, B). Based on CCK8 results, the cell viability of HCCLM3-sh-S100A11-1, 2 and Li-7-shS100A11-1, 2 was effectively suppressed compared to that in the control groups, respectively (Figure 5C, D). EdU incorporation assay results were consistent with the above results (Figure 5E, F). Conclusively, these data show that knockdown of S100A11 inhibits hepatoma cell proliferation.

In addition to cell proliferation, migration and invasion are also of crucial importance for cancer metastasis 26, 28, so we performed Transwell migration and invasion assay to detect the role of S100A11 in tumor metastasis. As the results showed, the amount of migrative and invasive cells in HCCLM3-sh-S100A11 group was significantly decreased compared with HCCLM3-sh-con group (Figure 5G). Similarly, knockdown of S100A11 inhibited Li-7 cell migration and invasion (Figure 5H). In a word, these results suggest that S100A11 knockdown affects not only cell proliferation but also cell migration and invasion.

### S100A11 activates AKT and ERK signaling pathways in hepatoma cells

**Figure 6:**
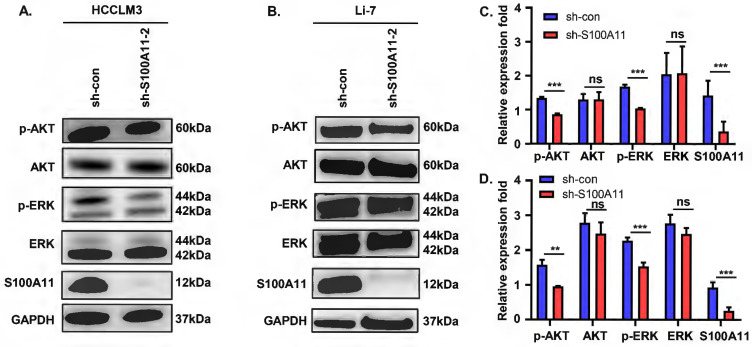
** S100A11 activates AKT and ERK signaling pathways. (A)** Effects of S100A11 knockdown on the phosphorylation of AKT and ERK in HCCLM3 cells examined by Western Blot. **(B)** Effects of S100A11 knockdown on the phosphorylation of AKT and ERK in Li-7 cells examined by Western Blot. **(C)** Statistical analysis of Western Blot assay for HCCLM3.** (D)** Statistical analysis of Western Blot assay for Li-7. ns: no significance, ** *P* < 0.01, *** *P* < 0.001.

These observations described above prompted us to explore the potential mechanisms. Previous studies showed that abnormal activation of AKT and ERK signaling pathways plays a vital role in tumor development and progression 29, 30. To analyze whether S100A11 activates AKT or ERK signaling pathway in hepatoma cells, we detected the expression and phosphorylation of key proteins in the two signaling pathways. The results of Western Blot showed that S100A11 knockdown in HCCLM3 and Li-7 cells decreased levels of AKT and ERK phosphorylation, while levels of total AKT and ERK remained unchanged (Figure 6A-D). Taken together, these data imply that S100A11 contributes to the activation of AKT and ERK signaling pathways.

## Discussion

HCC is the most common malignant tumor of the liver worldwide, and it is characterized by insidious onset, rapid progress, high degree of malignancy, and high rate of metastasis, bringing a great threat to global health, security, and the economy 3, 6, 8. China accounts for approximately half of all newly diagnosed HCC cases worldwide, with HBV infection being the predominant risk factor 4, 31. Despite significant efforts to improve HCC treatment, distant metastasis is still the main reason for the high mortality rate 7. As such, exploring the underlying molecular mechanisms of HCC development and metastasis and identifying effective biomarkers will help us to improve HCC diagnosis and treatment.

The S100 family comprises multiple calcium-binding proteins with two EF-hand domains 11. Distinct members, such as S100A4, S100A9, and S100A10, were reported to drive HCC development 32-34. Herein, we found that S100A11 was also overexpressed and related to worse prognosis and disease progression in HCC cohorts based on data from TCGA and GTEx databases. As expected, significantly higher S100A11 levels were observed for the first time in HCC patients than that in healthy groups. The ROC analysis showed good diagnostic performance of S100A11, and the sensitivity (97.1%) is sufficient to meet the clinical needs for HCC liquid biopsy. As we all know, AFP, a serum biomarker, is widely used in the clinical for HCC diagnosis with high specificity 35. But its sensitivity is low (just 58.8% in our study), resulting in a missing diagnosis of HCC frequently. Thus, biomarkers with high specificity and sensitivity are urgently needed. Delightfully, a novel finding in our study is that S100A11 and AFP nicely complement each other when used in combination, suggesting S100A11 may present as an effective diagnostic marker for conjunctional use with AFP in HCC diagnosis. Moreover, a defining feature of HCC is the high rate of metastasis, early detection of patients with metastasis is thus a key to reducing mortality from HCC 6. In our study, we revealed that S100A11 is superior to AFP in distinguishing patients with hematogenous metastasis from those without hematogenous metastasis, suggesting it could also be an effective prognostic indicator for HCC with poor clinical prognosis. In clinical, therapeutic approaches differ significantly between early-stage and advanced-stage HCC. Early HCC patients are often treated with surgery or liver transplantation, whereas patients with distant metastasis are usually treated with systemic therapy 7, 31. Therefore, S100A11 level can not only judge patients at risk of distant metastasis and poor prognosis but also help doctors choose the appropriate treatment approach.

The specific mechanism of tumorigenesis and metastasis is complicated and not well understood. Many studies have explored the role of S100A11 in tumorigenesis and its potential molecular mechanism in tumors. In most cancers, the expression level of S100A11 is increased and associated with tumor development and progression 14, 19, 25, 36. For example, Mab Meng et al. demonstrated that overexpression of S100A11 promotes the proliferation, migration, invasion and EMT of cervical cancer cells, and activates Wnt/β-catenin signaling pathway 19. Mingbing Xiao et al proved that S100A11 promotes human pancreatic cancer PANC-1 cell proliferation and is involved in the PI3K/AKT signaling pathway 36. Wenjing Li et al. indicated that S100A11 was overexpressed in serum of patients with epithelial ovarian cancer (EOC) and can be used as a biomarker for the diagnosis and treatment of ovarian cancer 37. On the other hand, S100A11 is considered to operate as a tumor suppressor in bladder cancer and the low expression of S100A11 is associated with patients with bladder cancer 21.

In order to explore the effects and underlying molecular mechanisms of S100A11 on HCC, *in vitro* models of S100A11 knockdown and overexpression in hepatoma cell lines were successfully constructed. We found that S100A11 knockdown cells grown in more dense colonies and enhanced cell-cell adhesion by up-regulating E-cadherin expression, while S100A11 overexpression cells grown more dispersedly by down-regulating E-cadherin expression, the results lead to a conclusion in agreement with a previous study investigating that S100A11 contributes to EMT process of hepatocytes 16. Regrettably, we did not detect any changes in expression of N-cadherin, the defined reason for the results was not clear. Besides, active tumor cell proliferation, migration, and invasion are prerequisites for the cascade of tumor metastasis 28. Our research demonstrated that knockdown of S100A11 reduced the proliferation, migration, and invasion of hepatoma cells, which provided sufficient evidence to another research reporting S100A11 as a key factor driving HCC 38. Simultaneously, abnormal AKT and ERK1/2 phosphorylation in HCC are hallmarks of tumor progression 29, 30, another promising finding in our study was that S100A11 knockdown inhibits AKT and ERK1/2 phosphorylation, which suggests that S100A11 is involved in the activation of AKT and ERK signaling pathways.

There are also some limitations in our study despite standard experimental procedures and strict implementation. Firstly, almost all HCC patients we enrolled had cirrhosis. So, we are unable to determine if S100A11 can distinguish between patients with liver cirrhosis and those with HCC. Secondly, large sample size studies are still needed to further validate these findings. Besides, the protein profile of the cells changes dramatically during EMT, but only two proteins (E-cadherin, N-cadherin) were detected in our study. Other marker proteins, such as Snail, Vimentin, Fibronectin, MMPs, and α-SMA still need to be investigated in this work. Finally, we only conducted experiments *in vitro* in this study, further *in vivo* experiments in animal models are required to confirm the functional and mechanistic contribution of S100A11 in HCC in the future.

Anyhow, our research revealed the function and underlying mechanism of S100A11 in HCC progression and metastasis and identified an effective predictor for HCC metastasis. Our study provides a theoretical and experimental basis for elucidating the mechanism of HCC development and new perspectives for HCC diagnosis and treatment.

## Conclusion

In summary, our studies demonstrate that S100A11 is significantly upregulated in HCC patients and associated with poor clinical outcomes. Subsequent assays imply for the first time that S100A11 may present as an effective circulating biomarker for conjunctional use with AFP in HCC diagnosis, and S100A11 outperforms AFP in identifying whether HCC patients possess hematogenous metastasis or not. Further experiments suggest that S100A11 promotes hepatoma cell metastasis by activating AKT and ERK signaling pathways and accelerating EMT progress. Overall, our findings highlight the clinical significance and underlying molecular mechanisms of S100A11 in HCC development and metastasis, casting a new light on the research of HCC metastasis and identifying a novel target for HCC diagnosis and treatment.

## Figures and Tables

**Table 1:** Characteristics of control donors and HCC patients

	Control	HCC
Case	22	34
Age (years)	48 ± 11	52 ± 9
Gender (male/female)	15 / 7	27/7
First diagnosis (Yes/No)	-	22/12
Cirrhosis (Yes/No)	-	34/0
Stage (I+II/III+ IV/unknown)	-	12/19/3
Non/Lymphatic/Hematogenous metastasis	-	18/4/12

**Table 2:** Sensitivity and specificity of S100A11, AFP, and S100A11+AFP using S100A11 optimal and AFP clinical cutoff values

	Cutoffs (ng/mL)	Sensitivity [95% CI]	Specificity [95% CI]
**Cohort 1**	**Control vs HCC**		
S100A11	0.82	97.06% [84.7-99.9]	54.55% [32.2-75.6]
AFP	20.00	58.82% [40.7-75.4]	100.00% [84.6-100]
S100A11+AFP	-	91.18% [76.3-98.1]	81.82% [59.7-94.8]
	
**Cohort 2**	**Non-hematogenous metastasis vs Hematogenous metastasis**		
S100A11	1.30	100.00% [73.5-100.0]	66.67% [38.4-88.2]
AFP	No diagnostic power		

## Abbreviations

PLC: primary liver cancer
HCC: hepatocellular carcinoma
S100A11: S100 calcium-binding protein A11
HBV: hepatitis B virus
FBS: fetal bovine serum
AFP: alpha fetoprotein
ROC: receiver operating characteristic
AUC: area under curve
EMT: epithelial-mesenchymal transition
